# *mappestRisk*: an R package for modelling and mapping risk of crop pest development based on their thermal biology

**DOI:** 10.7717/peerj.21428

**Published:** 2026-07-02

**Authors:** Darío San Segundo Molina, A. Marcia Barbosa, Antonio J. Pérez-Luque, Francisco Rodríguez Sánchez

**Affiliations:** 1 Departamento de Ciencias de la Vida, Universidad de Alcalá, Alcalá de Henares, Madrid, Spain; 2 Spatial Biology Lab (SBL), Universidade do Porto, Porto, Portugal; 3 Department of Ecology, Universidad de Granada, Granada, Spain; 4 Institute of Forest Sciences, National Institute of Agricultural and Food Research and Technology (INIA-CSIC), Madrid, Spain; 5 Departamento de Biología Vegetal y Ecología, Universidad de Sevilla, Sevilla, Spain

**Keywords:** Pest risk assessment, Integrated pest management, Biological invasions, Fundamental thermal niche, Forecasts, Thermal performance curves, Development rate

## Abstract

Under ongoing global warming and recent crop pest invasions, there is an urgent need to fill the gap between experimental research on pest thermal biology and applied forecasting tools that inform pest-management policies. The R package mappestRisk provides an accessible, open-source workflow that enables researchers, particularly those working in experimental pest biology, to forecast pest risk based on collected data on the thermal response of pest development rates. Built upon recent advances in open-source software development in the R environment, mappestRisk offers an accessible pipeline that spans from fitting performance curves for development rates data to generating broad-scale pest risk maps. Starting with user-provided temperature-dependent life-history dataset, the package fits nonlinear regression models and visualizes their thermal performance curves, with Akaike Information Criterion (AIC) scores and bootstrapped uncertainty ribbons. This allows users to select the most appropriate model based on biological, ecological and statistical criteria. The package then calculates thermal boundaries around the optimal region of the selected curve. These boundaries are used to extract climatic data for a user-defined spatial region or country and to map the number of months per year with optimal temperatures for pest development. The output consists of static or interactive raster maps that provide valuable insights for pest risk based on the known thermal biology of the target pest. This workflow contributes to making pest forecasts open, reproducible and accessible to the scientific community, while also providing relevant information for policy-making institutions and plant-protection organizations involved in crop-pest management.

## Introduction

Pests and pathogens cause substantial yield losses (around 20% on average) in major crops worldwide (Savary et al., 2019). Future warming is expected to increase the performance of several pests (Lehmann et al., 2020), leading to increased crop yield losses (Deutsch et al., 2018). Global warming will also heighten the risk of invasive pests as pest species extend their distribution polewards (Bebber, Ramotowski & Gurr, 2013) into novel regions. Anticipating these shifts requires forecasting pest performance and potential distributions under climate warming to better assess risk and support the development of adaptation policies and decision support systems aimed at safeguarding food security.

This need to understand how crop pests respond to temperature has motivated thousands of laboratory experiments on their thermal biology, examining how their biological rates vary with temperatures (*e.g.*, Liu & Tsai, 2000). More recently, international plant protection organizations, such as EPPO (https://gd.eppo.int/), CABI (https://www.cabidigitallibrary.org/journal/cabicompendium) and EFSA (https://www.efsa.europa.eu/en/topics/priority-pests), as well as research initiatives like VectorByte (https://www.vectorbyte.org) have compiled extensive data summarizing current knowledge on thermal biology of many pest species. Despite these efforts, a substantial gap remains between the experimental research on thermal biology of pests and applied forecasting tools used to inform pest management policies. These domains largely operate independently, with notable exceptions such as the International Potato Centre (CIP), which developed the Insect Life Cycle Modelling software (ILCyM, Sporleder et al., 2017) to guide management practices (see below).

Nonetheless, biological information on pest thermal responses has been recently used to map pest risk and generate prospective forecasts, particularly for species considered invasive in newly colonized regions (*e.g.*, Khadioli et al., 2014; Ponti et al., 2021; EFSA Panel on Plant Health (PLH) et al., 2023c; EPPO, 2024). For major global pests such as *Thaumatotibia leucotreta* (Meyrick), *Spodoptera frugiperda* (J.E. Smith), or tephritid fruit-flies like *Bactrocera dorsalis* (Hendel), extensive thermal biology data have enabled the development of demographic models that integrate the thermal dependence of multiple biological rates, leading to spatial–temporal forecasts (*e.g.*, Taylor et al., 2019; Gutierrez et al., 2021; EFSA Panel on Plant Health (PLH) et al., 2023a; Gilioli et al., 2023). However, because most pest species lack comprehensive experimental data across all their life-history traits, these models remain difficult to apply broadly in operational risk assessment tools.

In contrast, development rates variation across temperatures has been studied for decades (Chuine & Regniere, 2017; Rebaudo, Struelens & Dangles, 2018). These models have been widely used for predicting the seasonal emergence (*e.g.*, Hogg, Pitre & Anderson, 1982; Schmalensee et al., 2021) and the voltinism, or the number of generations per year (*e.g.*, Castex et al., 2020; Sampaio, Krechemer & Marchioro, 2021) to support Integrated Pest Management (IPM). One common approach relies on linear degree-day models (Campbell et al., 1974), which are particularly valuable for species with limited or no laboratory data, as they can be parameterized with only a few temperature treatments or even solely from field observations (*e.g.*, Lago et al., 2023). Nonetheless, linear degree-day models can yield biased predictions, especially under thermal regimes that frequently include high temperatures, because they cannot estimate upper developmental thresholds (Mirhosseini, Fathipour & Reddy, 2017) and/or cold at which they overestimate cold thresholds for development (Campbell et al., 1974) unless cut-offs are imposed (Moore & Remais, 2014), or under cold regimes, because they tend to overestimate cold developmental thresholds (Campbell et al., 1974).

Despite these limitations, spatial–temporal forecasts based on degree-day models remain widely used for anticipating pest impact, particularly for species with poorly characterized thermal biology. For example, the USA National Phenology Network (US-NPN, https://www.usanpn.org/data/maps/forecasts) provides degree-day forecasts for the seasonal emergence of multiple crop pests to guide IPM interventions. Similarly, the Degree-Days, Risk, and Phenological event mapping (DDRP) is a decision support tool that integrates thermal limits and degree-day models to predict the seasonal pest occurrence (Barker et al., 2020; Barker et al., 2023). The European Food Safety Authority (EFSA) frequently uses linear degree-day models as an initial approximation of climatic suitability based on potential voltinism (EFSA Panel on Plant Health (PLH) et al., 2023b; EFSA Panel on Plant Health (PLH) et al., 2023c; EFSA Panel on Plant Health (PLH) et al., 2024), often combined with correlative approaches such as CLIMEX (Early et al., 2022). Overall, degree-day models remain essential for mapping risk for multiple species, especially when biological data are scarce (Barker et al., 2020).

Nonlinear models, or thermal performance curves (TPCs) provide a biologically grounded alternative to linear degree-day, as development rates typically follow unimodal, left-skewed thermal responses (Régnier, Legrand & Rebaudo, 2022). Because TPCs fitted under constant, laboratory conditions can successfully predict performance in fluctuating environments (Bernhardt et al., 2018), they often yield more accurate predictions than linear models, particularly under thermal regimes that include temperatures above the optimum (the hot-decay region) or near the cold developmental threshold (Schmalensee et al., 2021). Simple TPC formulations typically include three parameters (Rebaudo & Rabhi, 2018), such as Brière-1 (Briere et al., 1999) and Lactin-1 (Lactin et al., 1995) equations. With appropriate starting values for the parameters, these models can be successfully fitted to development rate data when the number of temperature treatments exceeds the number of parameters (see documentation of the rTPC package; Padfield, O’Sullivan & Windram, 2025). TPCs represent an intermediate approach between simple linear degree-day models, optimal for species with limited data and rapid IPM decision-making, and demographic models that require experimental data for multiple biological rates. Therefore, this framework keeps the advantage of leveraging widely available experimental data on development rates while incorporating nonlinearity of biological rates in response to variable temperatures.

Open-source software development, particularly within the R environment (R Core Team, 2025), has greatly facilitated the fitting of TPCs and their application to ecological modelling, IPM and pest risk assessment. Several R packages provide tools for fitting nonlinear, least-squares regression models, including nlstools (Baty et al., 2024), nlme (Pinheiro et al., 2025), minpack.lm (Elzhov et al., 2023), nls2 (Grothendieck & R Core Team (nls), 2024) and the Bayesian, STAN-based brms package (Bürkner, 2017). Within this ecosystem, few packages have been developed for fitting TPCs for biological rates. The nls.multstart package (Padfield, O’Sullivan & Pawar, 2021) facilitates fitting nonlinear least squares regression models by iterating across multiple starting values for each parameter (Padfield, Matheson & Windram, 2025). The rTPC package facilitate provides tools to generate biologically informed starting values and to calculate ecologically meaningful parameters (*i.e.,* thermal traits or limits) derived from the fitted curves for ecological applications (Padfield, O’Sullivan & Windram, 2025). The devRate package (Rebaudo, Struelens & Dangles, 2018) offers functions to fit commonly used TPC equation models, a literature-based data set for finding starting values, and tools for predicting seasonal emergence and mapping voltinism (Rebaudo et al., 2025). Additionally, the bayesTPC package (Sorek et al., 2025) implements a Bayesian framework for fitting TPCs without the assumptions required by frequentist approaches. Similarly to DDRP, the Insect Life Cycle Modelling (ILCyM) software (Sporleder et al., 2017) is also built in R. It enables researchers to fit TPCs, simulate pest phenology, and generate risk maps that estimate the number of generations per year; establishment risk and an activity index based on population growth. Its graphical user interface further enhances accessibility for non-experts.

**Figure 1:**
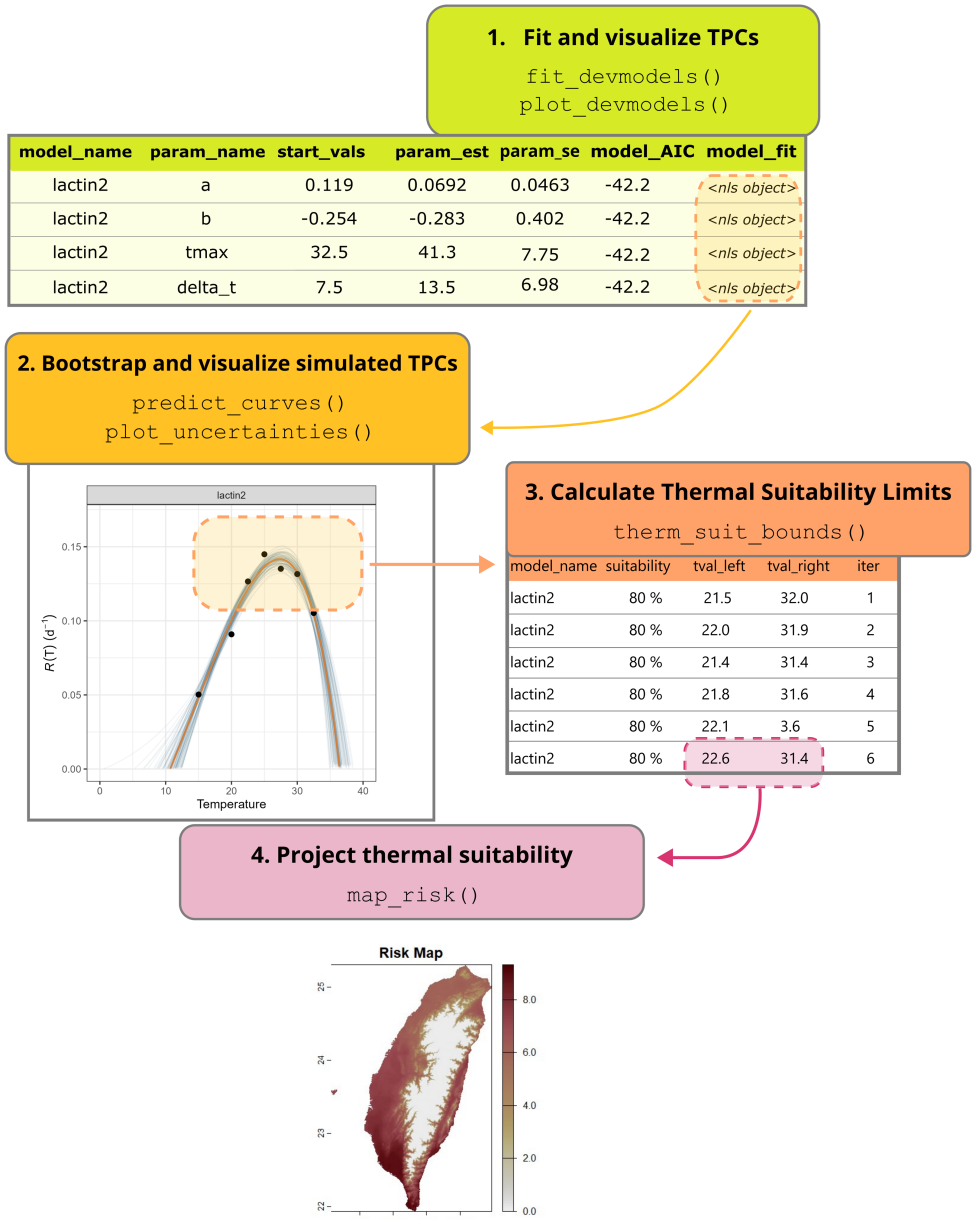
*mappestRisk* workflow of functions. The four steps accomplished by mappestRisk functions, from development rate data across temperatures to the generation of a forecast (the risk map). Note that the tables and figures are the same as those used for the example in the Results section.

Within this growing ecosystem, the mappestRisk R package (San-Segundo Molina et al., 2025) provides a reproducible, open-source and user-friendly pipeline to experimental scientists for modelling pest risk under climate variability (Fig. 1). It integrates the tidyverse suite for data handling (Wickham et al., 2019) and colourblind-friendly palettes *via* the khroma package (Frerebeau et al., 2025). Building on the functionalities of devRate and rTPC packages to obtain model equations and biologically informed starting values, the modelling framework of mappestRisk follows the workflow suggested for rTPC and nls.multstart (Padfield, O’Sullivan & Pawar, 2021), condenses it into few functions for model fitting, visualization, uncertainty propagation through bootstrapping, and extensions for mapping risk.

For spatial operations, mappestRisk uses the actively maintained geodata package (Hijmans et al., 2024) to automatically download temperature data from WorldClim (https://worldclim.org/data/worldclim21.html; Fick & Hijmans, 2017) and extract them over user-defined regions using the terra package (Hijmans et al., 2025) to produce exportable raster maps in .tiff format. These maps indicate the number of months per year with highly suitable temperatures for pest development (see ‘Material & Methods’).

mappestRisk is designed for users with basic R skills, offering a simple and unified workflow to fit TPCs for development rates and translate them into geographic forecasts for pest risk assessment, enabling prospective analyses across multiple pest species.

Relative to the existing R packages outlined above, mappestRisk (1) extends devRate with more TPC models and uncertainty propagation as suggested from rTPC package; (2) integrates rTPC and nls.multstart workflows into a simplified interface based on several functions for non-expert R users; and (3) provides functionality for elaboration of custom spatial forecasts for mapping pest risk, which can be completely integrated in data analysis routines. In addition, the package website (https://ecologyr.github.io/mappestRisk) includes extensive documentation for each function and several tutorials on how to use and extend the package.

In conclusion, mappestRisk helps bridge the gap ecological modellers working on pest applications, regional or international organizations requiring decision-support tools, and experimental researchers studying the thermal biology of crop pests. Integrating these efforts enables the production of custom, reproducible and interpretable forecasts as soon as biological data become available. This is particularly valuable for early risk assessment of pest species with limited thermal biology information, species entering new regions and potentially becoming invasive or species facing novel climatic conditions under mid-century warming scenarios. Moreover, the prospective risk maps generated with mappestRisk can serve as a preliminary guide for identifying where and when additional biological data are needed to support the development of more complex demographic and/or biophysical mechanistic models targeting specific threats at finer spatial and temporal scales.

## Material & Methods

### (1) Model fitting: nonlinear least-squares regression

Thermal performance curves describe the nonlinear dependence of several biological rates on temperature, particularly those associated with life-history traits (Amarasekare & Savage, 2012). Their shapes are typically unimodal (Huey & Stevenson, 1979; Angilletta, 2006) and, for development rates in arthropods, they are often left-skewed (Rebaudo & Rabhi, 2018; Régnier, Legrand & Rebaudo, 2022). Unlike linear degree-day models, fitting nonlinear regression models requires incorporating prior knowledge about model equations and parameter values. To support this process, the rTPC (Padfield, O’Sullivan & Windram, 2025) and devRate (Rebaudo et al., 2025) packages include multiple TPC equations (see Kontopoulos et al., 2024) for a systematic comparison), and provide tables and helper functions for finding suitable starting values for the parameters. The nls.multstart package (Padfield, Matheson & Windram, 2025) then offers a flexible nonlinear, least-squares framework that iterates across multiple starting values to efficiently fit these curves to experimental data.

To make this modelling framework more accessible to non-expert R users, we implemented the fit_devmodels function. The function requires as input a dataset containing values of temperatures (at least four different treatments for the simplest, three-parameter models, see section above) and their corresponding development rates. Users can select which TPC equations to fit from a predefined set (Table 1), preferably guided by prior knowledge of thermal responses in related organisms. These model equations can be inspected using the auxiliary function available_models. For each model equation, fit_devmodels function identifies appropriate starting values using functions from rTPC package and literature-based tables from devRate. Then, it iteratively fits nonlinear least-squares regression models using the nls.multstart package for each combination of equation and starting values. Models that fail to converge or show signs of false convergence (*e.g.*, extremely large parameter standard errors) are automatically discarded. The output is a table summarizing key statistical information for each fitted TPCs including model name, parameter estimates, standard error, AIC values stored model objects. This table is used for subsequent steps in the mappestRisk workflow (Fig. 1).

**Table 1:** TPCs models used in the mappestRisk package. It includes the standard names used in the functions, the equations and the source references. Detailed information for programming purposes can be easily accessed by typing available_models after loading the package.

Model name	Equation	Source reference
beta	$R= \frac{a{ \left( \frac{T-b+ \frac{c \left( d-1 \right) }{d+e-2} }{c} \right) }^{d-1}\cdot { \left( 1- \frac{T-b+ \frac{c \left( d-1 \right) }{d+e-2} }{c} \right) }^{e-1}}{{ \left( \frac{d-1}{d+e-2} \right) }^{d-1}\cdot { \left( \frac{e-1}{d+e-2} \right) }^{e-1}} $	Niehaus et al. (2012)
boatman	$R \left( T \right) ={r}_{max}\cdot (\mathit{sin}(\pi { \left( \frac{T-{T}_{min}}{T-{T}_{min}} \right) }^{a}))^{b}$	Boatman, Lawson & Geider (2017)
briere1	$R=a\cdot T\cdot \left( T-{T}_{min} \right) \cdot { \left( {T}_{max}-T \right) }^{ \frac{1}{2} }$	Briere et al. (1999)
briere2	$R=a\cdot T\cdot \left( T-{T}_{min} \right) \cdot { \left( {T}_{max}-T \right) }^{ \frac{1}{b} }$	Briere et al. (1999)
joehnk	$R={R}_{max} \left( 1+a\cdot \left( \left( {b}^{T-{T}_{\mathrm{opt}}}-1 \right) - \frac{lnb}{lnc} \left( {c}^{T-{T}_{\mathrm{opt}}}-1 \right) \right) \right) $	Jöhnk et al. (2008)
kamykowski	$R \left( T \right) =a\cdot (1-{e}^{-b\cdot \left( T-{T}_{min} \right) })\cdot (1-{e}^{-c\cdot \left( {T}_{max}-T \right) })$	Kamykowski (1986)
lactin1	$\begin{array}{@{}c@{}} \displaystyle R \left( T \right) ={e}^{\rho T}-{e}^{ \left[ \rho {T}_{max}- \frac{ \left( {T}_{max}-T \right) }{\Delta } \right] } \end{array}$	Lactin et al. (1995)
lactin2	$R \left( T \right) ={e}^{\rho T}-{e}^{ \left[ \rho {T}_{max}- \frac{ \left( {T}_{max}-T \right) }{\Delta } \right] }+\lambda $	Lactin et al. (1995)
mod_polynomial	$R \left( T \right) ={a}_{0}+{a}_{1}T+{a}_{2}{T}^{2}+{a}_{3}{T}^{3}+{a}_{4}{T}^{4}$	Rebaudo, Struelens & Dangles (2018)
mod_weibull	$R=a\cdot ( \frac{c-1}{c} )^{ \frac{1-c}{c} }( \frac{T-{T}_{\mathrm{opt}}}{b} +( \frac{c-1}{c} )^{ \frac{1}{c} })^{c-1}\cdot ex{p}^{-( \frac{T-{T}_{\mathrm{opt}}}{b} +( \frac{c-1}{c} )^{ \frac{1}{c} })^{c}}+ \frac{c-1}{c} $	Angilletta (2006)
oneill	$R \left( T \right) ={R}_{max}\cdot { \left( \frac{C{T}_{max}-T}{C{T}_{max}-{T}_{opt}} \right) }^{x}\cdot {e}^{x\cdot \frac{T-{T}_{opt}}{C{T}_{max}-{T}_{opt}} };$ $x= \frac{{w}^{2}}{400} \cdot { \left( 1+\sqrt{1+ \frac{40}{w} } \right) }^{2};$ $w= \left( {q}_{10}-1 \right) \cdot \left( C{T}_{max}-{T}_{opt} \right) $	Krenek, Berendonk & Petzoldt (2011)
pawar	$R= \frac{{R}_{Tref}\cdot ex{p}^{ \frac{-E}{k} \left( \frac{1}{T+273.15} - \frac{1}{{T}_{ref}+273.15} \right) }}{1+ \left( \frac{E}{{E}_{H}-E} \right) \cdot ex{p}^{ \frac{{E}_{h}}{k} \left( \frac{1}{{T}_{\mathrm{opt}}+273.15} - \frac{1}{T+273.15} \right) }} $	Kontopoulos et al. (2018)
ratkowsky	$R \left( T \right) ={ \left( a\cdot \left( T-{T}_{min} \right) \right) }^{2}\cdot { \left( 1-{e}^{b\cdot \left( T-{T}_{max} \right) } \right) }^{2}$	Ratkowsky et al. (1983)
schoolfield	$R= \frac{{R}_{Tref}\cdot ex{p}^{ \frac{-E}{k} \left( \frac{1}{T+273.15} - \frac{1}{{T}_{ref}+273.15} \right) }}{1+ex{p}^{ \frac{{E}_{L}}{k} \left( \frac{1}{{T}_{L}} - \frac{1}{T+273.15} \right) }+ex{p}^{ \frac{{E}_{H}}{k} \left( \frac{1}{{T}_{H}} - \frac{1}{T+273.15} \right) }} $	Schoolfield, Sharpe & Magnuson (1981)
thomas	$R=a\cdot ex{p}^{b\cdot T} \left( 1-( \frac{T-{T}_{ref}}{ \frac{c}{2} } )^{2} \right) $	Thomas et al. (2012)
wang	$R \left( T \right) = \frac{K}{1+{e}^{-r\cdot \left( T-{T}_{0} \right) }} \cdot \left( 1-{e}^{- \frac{T-{T}_{L}}{aa} } \right) \cdot \left( 1-{e}^{- \frac{{T}_{H}-T}{aa} } \right) $	Wang, Lan & Ding (1982)

TPC model selection should be guided primarily by biological and ecological reasoning rather that purely statistical criteria (Quinn, 2017). No single TPC equation consistently performs best across species or datasets (Kontopoulos et al., 2024), so comparing multiple fitted curves using expert knowledge is essential to minimize arbitrariness in model selection. Specialists in thermal biology and ecology of the target pest, as potential users of the package, can use their expertise to identify which fitted TPCs best reflects the expected response across the thermal regimes experienced by field populations (Khelifa et al., 2019; Schmalensee et al., 2021). To support this process, the plot_devmodels function provides quick visualizations of predictions from the fitted TPCs using the table generated by fit_devmodels.

### (2) Propagating parameter uncertainty with bootstrap

Forecasting involve multiple sources of uncertainty, including model structure, parameter uncertainty, and errors in response or predictor variable (Simmonds et al., 2022). Propagating this uncertainty when modelling pest performance through TPCs and their subsequent geographical predictions is critical to avoid biased forecasts (Woods et al., 2018).

To address this, the predict_curves function in mappestRisk simulates new TPCs using bootstrap with residual resampling, following the approach implemented in the rTPC package (Padfield, O’Sullivan & Windram, 2025; see also Puth, Neuhäuser & Ruxton, 2015). Residual resampling was chosen over case resampling because predictor values laboratory experiments are typically controlled, and the limited representation of the hot-decay region of the curves in many datasets would make case resampling refits unstable. Users can select converged models to bootstrap and specify the number of iterations (defaults to 100). Internally, the function extracts residuals and fitted values from the selected TPC model and generates new observations for each bootstrap iteration *i* (*y*_*i*_) as:

\begin{eqnarray*}{y}_{i}=\hat {y}+{e}_{i}, \end{eqnarray*}

where $\hat {y}$ represents the fitted values and *e*_*i*_ the resampled residuals. This produces *n*-resampled data sets, each used to implicitly refit a new TPC *via* fit_devmodels. For the subset of models that adequately converged (*k* bootstrapped curves), predict_curves computes predictions along temperature sequence at 0.1 °C intervals, spanning from 20 °C below the minimum to 15 °C above the maximum temperature in the original data set. The resulting *k* simulated curves are then used to propagate parameter uncertainty.

The function outputs a table containing all simulated TPCs, which can be used for visualization and for calculating ecologically meaningful parameters (*e.g.*, thermal traits). The plot_uncertainties function provides an intuitive visualization of these results, displaying the originally fitted TPC as a central curve and the *k*-bootstrapped curves as an uncertainty ribbon (see Fig. 1).

### (3) Calculate thermal suitability boundaries

The risk index implemented in mappestRisk is inspired by the previous work by Taylor et al. (2019) (see also Mordecai et al., 2017; Pardo-Araujo et al., 2024; Shocket et al., 2025). These developed a TPC-based model describing the thermal dependence of citrus greening disease transmission by the Asian citrus psyllid, *Diaphorina citri* Kuwayama, 1908. Because this approximates the fundamental thermal niche of the species for pathogen transmission (Simon & Amarasekare, 2024), they projected the number of months per year with optimal temperatures for disease spread across major citrus-growing regions. This index, expressed as the number of high risk months, provides a direct and easily interpretable measure that supports policy making by reducing uncertainty in risk communication (Simmonds et al., 2022).

Development rate TPCs play a central role in shaping the thermal dependence of fitness (*e.g.*, as population growth rates) and, therefore, the fundamental thermal niche of ectotherms (Amarasekare & Savage, 2012; Pawar et al., 2024). Building on this idea, we propose a simplified framework to estimate how many months per year offer optimal temperatures for maximizing pest development. Although fitness-related traits such as intrinsic rates of increase would be ideal for this purpose (Amarasekare & Coutinho, 2013), such data are unavailable for most crop pests, whereas development rate data are widely accessible. Our approach follows the logic of voltinism maps (*e.g.*, EFSA Panel on Plant Health (PLH) et al. 2023a; EFSA Panel on Plant Health (PLH) et al. 2023b; EFSA Panel on Plant Health (PLH) et al., 2024), but incorporates nonlinear thermal responses, uncertainty propagation, and a directly interpretable risk metric.

To compute this index, the therm_suit_bounds function uses the output from predict_curves to estimate the thermal limits (or suitability boundaries) defining the temperature range where development is maximized. These thermal limits can be customized by the user; by default, the function uses the 75th percentile of the curve height (*i.e.,* 75% of the maximum predicted rate *R*_*max*_). Then, it identifies the two temperatures surrounding the TPC peak at which predicted rates are equal 0.75*R*_*max*_. When multiple simulated TPCs are available from bootstraps, therm_suit_bounds returns a table of w thermal limits for each simulated curve, thereby incorporating parameter uncertainty into a distribution of thermal suitability boundaries.

### (4) Project thermal suitability

Given the fitted and simulated TPCs and their corresponding thermal suitability boundaries, mappestRisk can automatically generate raster maps showing the number of suitable months for pest development using the map_risk function. This function operates in three steps:

I. Climatic data extraction: if the user does not temperature rasters, map_risk automatically downloads monthly temperature layers from the WorldClim historical data set (https://worldclim.org/data/worldclim21.html) using the geodata R package (Hijmans et al., 2024). The resulting raster stack contains 12 layers (one per month) at user-defined spatial resolution (default to 2.5 arcminutes, ∼4.6 km at the equator). These data are then cropped and masked to the target region, which can be specified as a country name, a spatial extent or a vector object.
II. Spatial operations: using the terra package, map_risk processes each monthly layer to generate a logical raster in which cells receive a value of 1 when the monthly mean temperature falls within the range defined by the thermal suitability boundaries from therm_suit_bounds, and 0 otherwise. Summing these 12 layers yields the annual number of highly suitable months for development. To propagate parameter uncertainty into the spatial forecasts (Woods et al., 2018), this procedure is repeated for each iterated set of thermal boundaries derived from bootstrap-generated TPCs. The final output is a two-layer raster: a risk layer, representing the mean number of suitable months acrss all iterations; and an uncertainty layer, representing the standard deviation across iterations.
III. Visualization: the resulting raster can be exported for further analysis visualized directly through map_risk. The function can produce a static plot with panels for the risk and uncertainty layers, or an interactive map *via* a leaflet-HTML object generated through terra.

## Results

### Package installation and basic usage example

We illustrate the suggested workflow of mappestRisk using its included aphid data set, which consists development rate measurements for the aphid *Brachycaudus schwartzi* Börner, (1931), across seven constant temperatures under laboratory conditions (Satar & Yokomi, 2002).

The stable version of mappestRisk can be installed from CRAN (install.packages (“mappestRisk”)) and the development version from GitHub (remotes::install_github (“EcologyR/mappestRisk”)). Documentation, examples and vignettes (including more advanced use cases such as mapping potential exclusion by heat stress, seasonal and rate summation forecasts, and interactive maps) are available in the package website (https://ecologyr.github.io/mappestRisk/).

After loading the package (library(mappestRisk)) and the aphid data (data(“aphid”)), for illustrative purposes, we first fit all available TPC models using:

aphid_tpcs <- fit_devmodels(temp = aphid$temperature,

dev_rate = aphid$rate_value,

model_name = “all”)

The aphid_tpcs object is a tibble (data frame) with eight columns summarizing all models that converged successfully with relevant statistical information (see ‘Material & Methods’).

The fitted curves can be visualized with:

plot_devmodels(temp = aphid$temperature,

dev_rate = aphid$rate_value,

fitted_parameters = aphid_tpcs,

species = “Brachycaudus schwartzi”,

life_stage = “Nymphs”)

**Figure 2:**
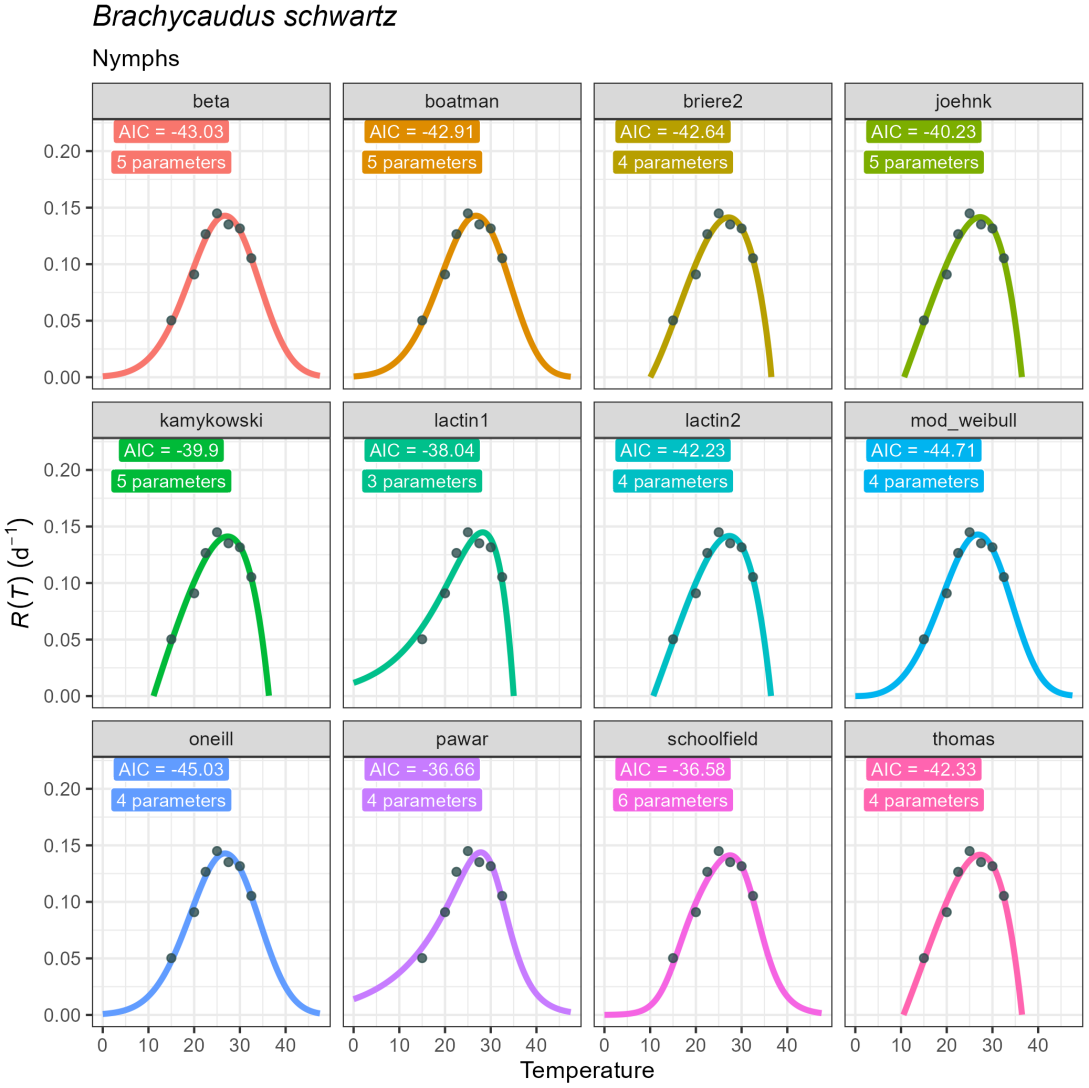
Predictions of the thermal performance curves fitted to users’ data. The graph of these fitted curves is obtained through the plot_devmodels function, and the thermal performance curves are fitted with the fit_devmodels function.

This produces the panel shown in Fig. 2.

To obtain thermal limits for mapping, users must first generate predictions with predict_curves, regardless of whether uncertainty is propagated. In the example below, we simulate 100 bootstrapped for two selected models:

preds_boots_aphid <- predict_curves(temp = aphid$temperature,

dev_rate = aphid$rate_value,

fitted_parameters = aphid_tpcs,

model_name_2boot = c(“lactin2”, “thomas”),

propagate_uncertainty = TRUE,

n_boots_samples = 100)

The output contains predicted development rates across temperatures, along with model name, the iteration ID, the curve type (estimated or simulated). These curves can be visualized with:

plot_uncertainties(temp = aphid$temperature,

dev_rate = aphid$rate_value,

bootstrap_tpcs = preds_boots_aphid,

species = “Brachycaudus schwartzi”,

life_stage = “Nymphs”)

**Figure 3:**
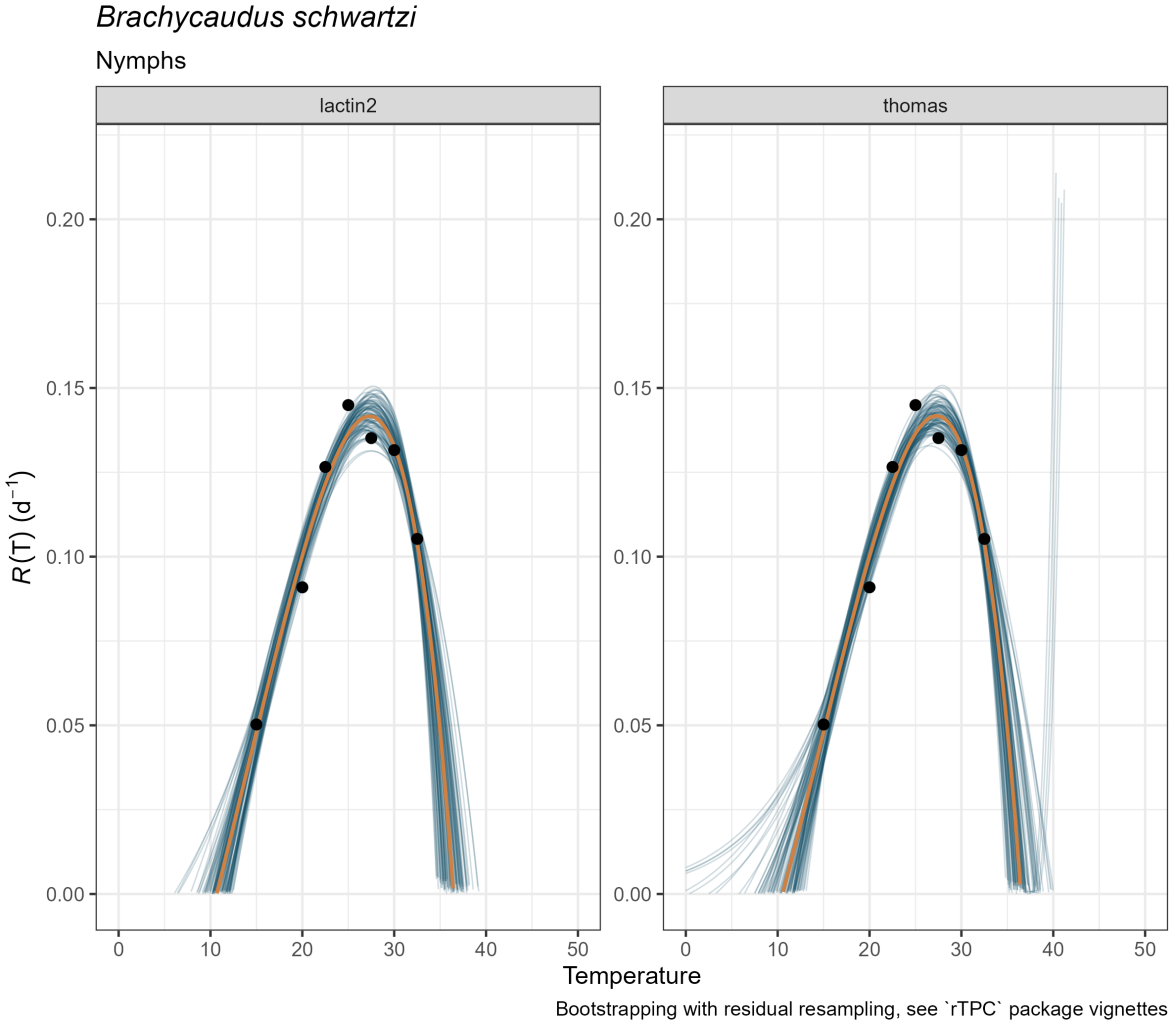
Simulated, bootstrapped thermal performance curves. The graph of the curves is obtained with the plot_uncertainties function, and the bootstrapped curves are obtained with predict_curves function.

This code produces Fig. 3.

For risk mapping, we select the lactin2 model and compute thermal suitability limits using the top 20th of the curve, following Taylor et al. (2019):

aphid_bounds <- therm_suit_bounds(preds_tbl = preds_boots_aphid,

model_name = “lactin2”,

suitability_threshold = 80)

The resulting tibble (data frame) contains, for each bootstrap iteration, the left and right thermal suitability boundaries, the predicted rate at those temperatures, and metadata such as the suitability threshold, iteration ID and model name.

Finally, we use these boundaries to forecast and map the number of highly suitable months for development of *B. schwartzi* in Taiwan, as an illustrative example. If no temperature raster is provided, map_risk function automatically downloads WorldClim data and masks it to Taiwan:

aphid_risk_raster <- map_risk(t_vals = aphid_bounds,

region = “Taiwan”,

path = tempdir())

**Figure 4:**
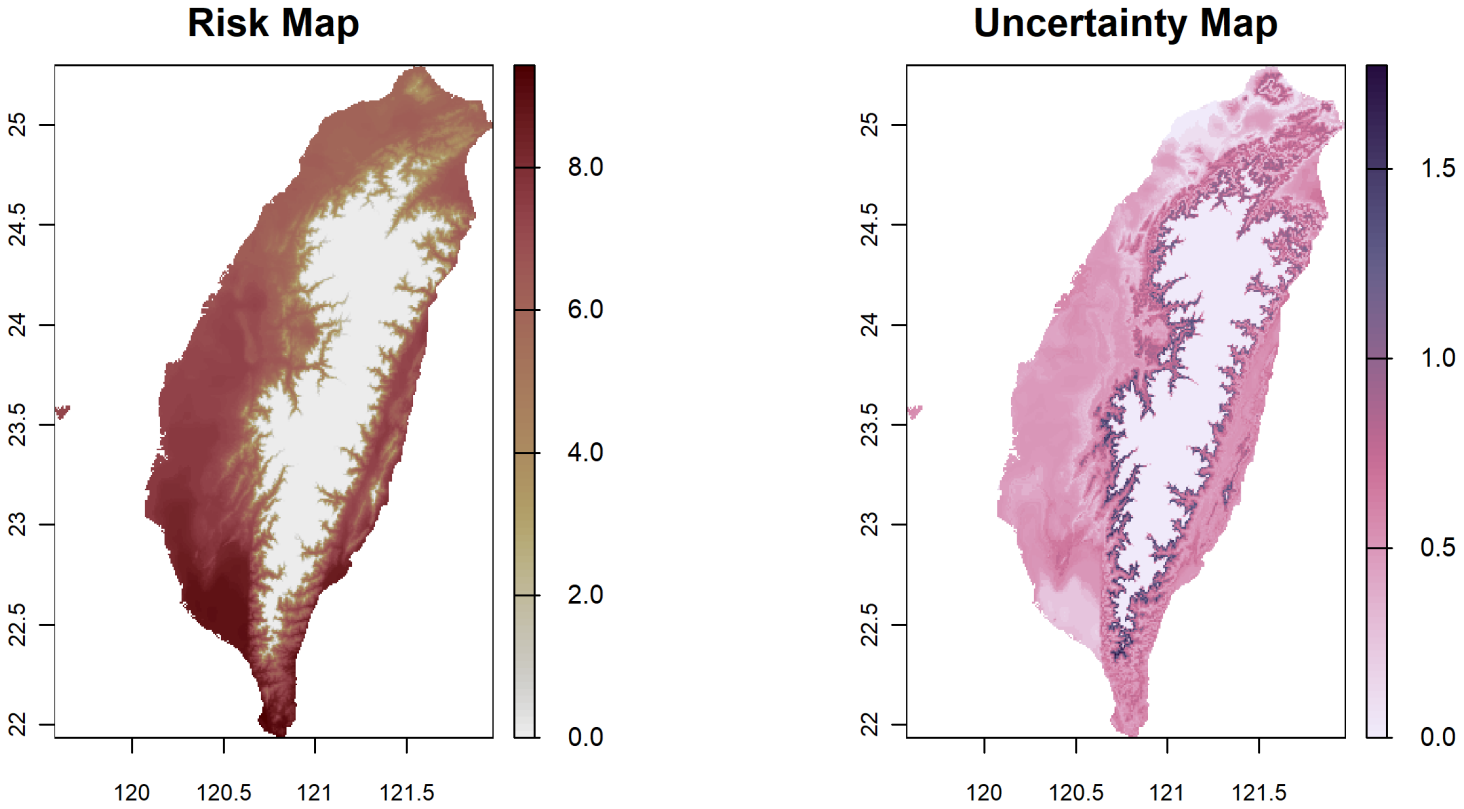
Risk and uncertainty maps obtained from *map_risk* function. The left side shows the risk map (*i.e.,* showing the number of months with highly suitable temperatures for the pest development, as obtained with previous functions of the package). The right side map shows the standard deviation of the index calculated for the risk map, which indicates the parameter uncertainty of the risk forecast.

The resulting raster (stored in aphid_risk_raster) can be exported for GIS processing. By default, the function also returns a companion plot (Fig. 4). In this example, the map indicates that most coastal regions of Taiwan can experience 7 months per year (±1 month) with optimal temperatures for the development of *B. schwartzi*.

## Discussion

### Limitations and future development

mappestRisk uses interpolated data (coming from meteorological stations) by default. Because those macroclimatic temperatures often differ from the operative or body temperatures experienced by pest organisms, forecasts based on coarse climatic data may lead to biased predictions of performance under field conditions, especially at fine spatial or temporal scales (Mosedale et al., 2024). Similarly, our risk metric, based on the accumulation of performance near the thermal optimum, can be biased when input temperatures are aggregated, as this ignores the distribution of temperatures around the mean (Bütikofer et al., 2020). Consequently, mappestRisk forecasts generated with default WorldClim data (*i.e.,* at coarse spatial and temporal scales) should not be interpreted at local scales (Bütikofer et al., 2020), a limitation shared with other decision-support tools such as DDRP, US-NPN and ILCyM.

Using microclimatic conditions or mechanistic estimates of operative temperatures through biophysical modelling (*e.g.*, *via* NicheMapR; Kearney & Porter, 2020) would provide more realistic predictions at fine scales and help avoid biases arising from nonlinear thermal responses applied to coarse-resolution climate data (Bütikofer et al., 2020). At present, the map_risk function does not accept daily or hourly temperature data directly, but users can provide their own (micro)climatic layers to achieve higher resolution forecasts (see tutorial on the package website). Future package versions will allow predictions based on more flexible user-provided temperature datasets, facilitating integration with mechanistic biophysical models. These updates, developed under the package’s maintenance program, may also incorporate additional features, such as new TPC equations, extensions for modelling other biological rates, and more flexible climatic suitability indices, as illustrated in the package website articles.

## Conclusions

The developmental thermal responses of major crop pests revealed in experimental biology can be used to inform the assessment and mapping of pest distribution and risk. mappestRisk integrates recent advances in open-source software with new methodological and theoretical developments in thermal biology and ecology. The package is designed to facilitate spatial–temporal forecasting based on physiological traits of pest species for applications such as biological invasions, pest risk assessment and phenological predictions.

Its integration with other packages within the R environment makes the workflow open, reproducible and accessible to researchers across disciplines. This integration has the potential to help bridge the gap between experimental researchers studying pest thermal biology, ecological modellers in global change ecology and administrative institutions for policy making. The mappestRisk package is available on CRAN (San-Segundo Molina et al., 2025), and a development version under active maintenance hosted on GitHub (https://github.com/EcologyR/mappestRisk).

## Additional Information and Declarations

### Data Availability

The following information was supplied regarding data availability: The data and code to install the mappestRisk package (v.-0.1.2) and reproduce the example is available on CRAN: https://doi.org/10.32614/CRAN.package.mappestRisk.
